# A novel non-invasive brain stimulation technique: “Temporally interfering electrical stimulation”

**DOI:** 10.3389/fnins.2023.1092539

**Published:** 2023-01-27

**Authors:** Wanting Guo, Yuchen He, Wenquan Zhang, Yiwei Sun, Junling Wang, Shuang Liu, Dong Ming

**Affiliations:** ^1^Academy of Medical Engineering and Translational Medicine, Tianjin University, Tianjin, China; ^2^Department of Biomedical Engineering, College of Precision Instruments and Optoelectronics Engineering, Tianjin University, Tianjin, China; ^3^Tianjin International Joint Research Center for Neural Engineering, Tianjin, China

**Keywords:** temporally interfering electrical stimulation, transcranial electrical stimulation, deep brain stimulation, interferential current, electrophysiological mechanism, therapeutic applications

## Abstract

For decades, neuromodulation technology has demonstrated tremendous potential in the treatment of neuropsychiatric disorders. However, challenges such as being less intrusive, more concentrated, using less energy, and better public acceptance, must be considered. Several novel and optimized methods are thus urgently desiderated to overcome these barriers. In specific, temporally interfering (TI) electrical stimulation was pioneered in 2017, which used a low-frequency envelope waveform, generated by the superposition of two high-frequency sinusoidal currents of slightly different frequency, to stimulate specific targets inside the brain. TI electrical stimulation holds the advantages of both spatial targeting and non-invasive character. The ability to activate deep pathogenic targets without surgery is intriguing, and it is expected to be employed to treat some neurological or psychiatric disorders. Recently, efforts have been undertaken to investigate the stimulation qualities and translation application of TI electrical stimulation *via* computational modeling and animal experiments. This review detailed the most recent scientific developments in the field of TI electrical stimulation, with the goal of serving as a reference for future research.

## 1. Introduction

Neuromodulation technology has emerged as a promising diagnostic and therapeutic technique in the past three decades (Ramirez-Zamora et al., 2017; Denison and Morrell, 2022). Unlike pharmacological treatments, neuromodulation therapies frequently employ physical or chemical methods to regulate the excitability of specific neural networks, which have been found effective in the treatment of neuropsychiatric disorders such as Parkinson’s disease, epilepsy, obsessive compulsive disorder and so on, offering novel treatment options for patients. Consequently, neuromodulation technology has demonstrated tremendous potential for treating neurodegenerative and psychiatric disorders (Di Pino et al., 2014; Boes et al., 2018).

The significant modalities of neuromodulation technologies include invasive and non-invasive brain stimulation techniques. Deep brain stimulation (DBS) is one of the advanced invasive neuromodulation modalities, this technique can directly intervene the pathological neural circuits by implanting electrodes in specific brain targets (Ramirez-Zamora et al., 2017). As a highly focal invasive neuromodulation approach, DBS has helped over 100,000 patients with movement abnormalities such as Parkinson’s disease, tremor, and dystonia (Lozano et al., 2019). And it also shows great potential in the treatment of neuropsychiatric diseases such as Alzheimer’s disease and treatment-resistant depression (Aum and Tierney, 2018). However, DBS remains an invasive technique that carries hazards such as intracerebral hemorrhage and infection, which may limit its practical application (Rossi et al., 2018). In contrast to invasive modulation, transcranial electrical stimulation (TES) and transcranial magnetic stimulation (TMS) are two non-invasive techniques commonly used in the clinic, which utilize electrodes or coils to apply electric or magnetic forces to the human scalp, resulting in both acute and neuro-plastic alterations in cortical excitability (Rawji et al., 2020). TES and TMS can be used for the treatment of epilepsy, stroke, schizophrenia and depression (Camacho-Conde et al., 2022). These non-invasive techniques hold the advantages of safety, well-tolerated, cost-effective, and easy to operate (Yavari et al., 2018). Nevertheless, due to the complicated structure of human brains, the electric and magnetic fields often decline dramatically as depth increases, leading to the low spatial resolution of most non-invasive brain stimulation modalities (Voroslakos et al., 2018).

Overall, it is significant and necessary to develop novel neuromodulation techniques with both high spatial resolution and non-invasive character (Caulfield and George, 2018; Liu et al., 2021). In Grossman et al. (2017) proposed a novel non-invasive brain stimulation technique—“temporally interfering” (TI) electrical stimulation. It is worth noting that TI electrical stimulation can stimulate deep brain targets without surgery, holding the advantages of both spatial targeting and non-invasive character. With the ability to stimulate deep pathogenic areas, TI electrical stimulation has the potential to treat neuropsychiatric disorders. However, this technique is still in its early stages, with several continuing efforts to investigate it further in terms of computational models, animal research, and human trials (Bouthour et al., 2017; Dmochowski and Bikson, 2017). This review summarizes the current research progress of TI electrical stimulation, aiming to give a reference for future research and further facilitate development and application. The main contents of this review: (1) The fundamental physics and potential neural mechanisms of TI electrical stimulation; (2) Effects of TI electrical stimulation on motor function and its application in clinical diseases treatment; (3) Various optimization schemes for TI electrical stimulation, including stimulation electrodes, parameters, and hardware, etc.

## 2. Current progress of TI

### 2.1. Origin and principles

In the 1950s, Austrian scientist Hans Nemen proposed a type of electrical stimulation therapy for peripheral stimulation called interferential currents (IFC), which used two medium-frequency sinusoidal currents of different frequencies (usually 4000 Hz and 4000 Hz ∼ 4250 Hz) to interfere with each other, and then produced a low-frequency interference current of 0∼250 Hz for stimulation at the intersection of the two currents (Goats, 1990). Considering that high-frequency current can easily penetrate human tissue while low-frequency current has better stimulating effects, IFC is able to inject more currents into tissues without reaching pain thresholds in the skin. This approach is now extensively applied in the treatment of disorders such as chronic pain in the muscle or back, urine incontinence, and constipation (Fuentes et al., 2010; Facci et al., 2011; Almeida et al., 2018).

**FIGURE 1:**
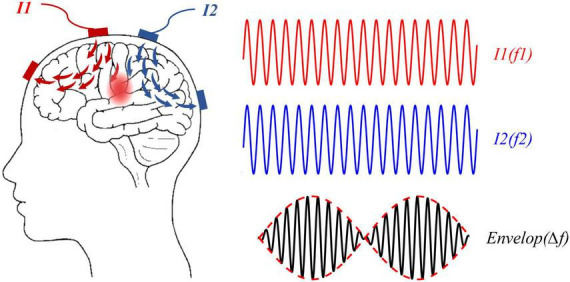
Temporally interfering (TI) electrical stimulation applied two high-frequency sinusoidal currents, I1 (red line) and I2 (blue line), at a small frequency difference (△f) to a human brain, which could generate a modulation waveform (solid black line), and the envelope of the modulated waveform was a low-frequency waveform (red dotted line) at the difference frequency △f.

In Grossman et al. (2017) creatively utilized the principle of interference to the brain and developed TI electrical stimulation. The principle of TI electrical stimulation was shown in Figure 1, two high-frequency sinusoidal waveforms (f1 and f2) of slightly different frequency were applied to the brain, then a low-frequency envelope waveform at △f (f1-f2) would produce inside the brain, and hence functions as a direct △f alternating current modulation. In the first *in vivo* study, TI electrical stimulation effectively activated neurons in the hippocampus of mice using electrodes attached to the skull. It was the first time that non-invasive and focal stimulation in deep brain areas was validated, paving the way for a new direction in the field of brain stimulation. Given the importance of deep pathological locus stimulation in the treatment of neurological and psychiatric disorders such as Parkinson’s disease, stroke, depression, and obsessive-compulsive disorder, the prospect of targeted stimulation without surgery is appealing (Tye and Deisseroth, 2012; Harmsen et al., 2020). That is why TI electrical stimulation has received so much interest in the field of neuroscience since its inception.

### 2.2. Electrophysiological mechanisms

Grossman’s study assumed the working mechanism of TI electrical stimulation as low-pass filtering of neural membranes (Grossman et al., 2017). However, this hypothesis was challenged with the deepening of research. Some studies had found that the fundamental physics of TI electrical stimulation involved an ion-channel-mediated current rectification mechanism (Mirzakhalili et al., 2020). And some other studies also evidenced its neuromodulatory effects may be related to subthreshold modulation (Howell and McIntyre, 2021).

#### 2.2.1. Fundamental physics

Previous research on TI electrical stimulation relied on the neural membrane’s intrinsic low-pass filtering property (Grossman et al., 2017). The outer membrane’s parallel leak conductance and capacitance constitute the equivalent of a filter that attenuates responses to high-frequency inputs (Hutcheon and Yarom, 2000). In specific, a 10 Hz interference current successfully drove neuronal spiking in anesthetized live mice, whereas a sinusoidal current of 2 kHz failed to trigger neurons to fire, according to Grossman et al. (2017). As research has evolved, the understanding of the low-pass filtering characteristics has been broadened. Karimi et al. (2019) exploited axon model to investigate the responses of the neurons to TI-induced electric fields, and they found that the axon does not follow high-frequency components, but rather the envelope waveform of the input signal by demodulating the input signal in the axon model. Some researchers believed that the fundamental low-pass filtering assumption was quite idealistic (Mirzakhalili et al., 2020). Although the envelope waveform of TI electrical stimulation is a low-frequency current produced by the superposition of two kilohertz magnitude currents, this waveform, in fact, only contains high-frequency components, which cannot be extracted by low-pass filtering characteristics of neurons alone. Following that, more research was performed to investigate the fundamental physics of TI electrical stimulation. Mirzakhalili et al. (2020) established an axon model for analysis using the standard Hodgkin-Huxley formula. As a cornerstone in the field of computational neuroscience, Hodgkin-Huxley formula can be used to investigate the neuronal function and further provide accurate reflection of single neuron (Petousakis et al., 2022). The findings indicated that TI stimulation necessitates an ion-channel-mediated signal rectification process in order to extract low-frequency envelope waveforms and activate neural activity. They also revealed that the rectification process is related to the specific gating properties of fast Na^+^ channels after that. In addition, this work also indicated that TI electrical stimulation’s source sinusoidal currents are responsible for high-frequency conduction block in the off-target areas, high-frequency electrical stimulation blocks or inhibits the propagation of action potentials along the axon.

To sum up, the non-responsiveness of neurons in the superficial and off-target regions may be related to the effect of conduction block, according to these observations. It might, however, cause undesired side effects and restrict the therapeutic applicability of TI electrical stimulation (Mirzakhalili et al., 2020). Future studies should consider the effects of conduction block in the tentative design of TI electrical stimulation.

#### 2.2.2. Possible mechanisms

In order to better understand the neuromodulatory effects of TI electrical stimulation, recent studies also began to explore other underlying mechanisms *via* neuron and axon models. Howell and McIntyre (2021) exploited the axon and neuron models to evaluate suprathreshold and subthreshold modulation with TI electrical stimulation, respectively. The results demonstrated that axons failed to initiate action potentials at 10 mA or less and became quiescent along with the inactivation of sodium channels. Consequently, TI electrical stimulation cannot activate the most excitable axonal elements when the stimulation intensity is ≤10 mA. On the contrary, TI electrical stimulation could modulate the spiking activity indirectly by facilitating the phase synchronization of neuron models at 2 mA or less, which was similar to transcranial alternating current stimulation (tACS) (Tavakoli and Yun, 2017). These results demonstrated that the mechanism of TI electrical stimulation might refer to the subthreshold modulation of neurons.

Furthermore, a previous study applied TES on rats and reported that an intracerebral voltage gradient of at least 1 V/m was required to affect neuronal spiking, whether the alternating currents were applied subcutaneously or transcutaneously (Voroslakos et al., 2018). Some computational modeling studies also investigated the electric field intensity of human brains generated by TI electrical stimulation. To deliver TI currents to specific brain targets, Lee et al. (2020) employed three finite element head models to determine the optimized electrode configurations and injection currents. The results found that the maximum electric field intensity at the targeted right hippocampus was only 0.38 V/m in a human finite element head model. Additionally, a realistic human head models study by Rampersad et al. (2019) indicated the maximum electric field generated in the human brain at any position and direction is less than 0.8 V/m when applied a stimulation current of 2 mA. They also found that over 150 mA of scalp currents in human brains would be required for acute neuronal modulation with TI electrical stimulation. In a word, the TI-induced electric fields applied in multiple studies are far from triggering action potentials in brain structures, suggesting the subthreshold neuromodulation rather than direct activation of neurons under conventional TI electrical stimulation.

On the other hand, Cao and Grover (2018) explored the stimulatory effects of TI stimulation on different neurons, and they found that classical Hodgkin-Huxley neurons and neocortical pyramidal neurons responded, and yet PV neurons did not. Therefore, TI electrical stimulation may not be effective on all neuron types, indicating the possibility of second-order network-level effects of this approach. In another study, the researchers studied the selective electrical stimulation of myelinated nerve fibers of TI electrical fields (Wang and Dokos, 2021). The results showed that TI electrical stimulation could selectively stimulate myelinated nerve fibers in nerve bundles. Besides, the myelinated fibers were more easily activated by TI electrical stimulation than unmyelinated fibers. Most specifically, some studies revealed that the collective activities of neuronal networks demonstrated more sensitivity to electric fields compared to single neurons (Deans et al., 2007; Bernardi and Lindner, 2019). According to the above studies, the selective activation of neurons and nerve fibers indicated that TI electrical stimulation might work by brain neural networks to some extent.

### 2.3. Developments and applications

In most current studies, animal models, as well as human trails, were exploited to study the application of TI electrical stimulation. Researchers had not only found the effectiveness of TI electrical stimulation on motor function (Ma et al., 2021), but also explored several feasible clinical applications, such as localization of epileptogenic zones, and respiratory stimulation (Collavini et al., 2021; Sunshine et al., 2021).

#### 2.3.1. Motor function modulation

Herein demonstrated the modulation of TI electrical stimulation on the motor cortex in both rodents and humans’ brain. Grossman et al. (2017) observed the periodic movement of the forepaw and whiskers in mice when applied TI currents on the motor cortex. Subsequent studies further explored the regulation effects of TI electrical stimulation. For instance, Zhang et al. (2022) found TI electrical stimulation could activate the primary motor cortex in rats. They used cranial electrodes to stimulate the motor cortex of living rats and observed the movement of the rat’s forepaw and the changes of the electromyography (EMG) similarly. Moreover, Song S. et al. (2021) applied TI electrical stimulation to the superior colliculus of mice, which is an important midbrain structure involved in sensorimotor translation. After the analysis of the recording Ca^2+^ signals and eye movement amplitudes, researchers found the neural activity in deep layers of the superior colliculus would cause eye movements in mice during TI electrical stimulation. Whereas no similar eye movements or neural activity were observed in mice when applying the same intensity and frequency of tACS for stimulation, suggesting that TI electrical stimulation provided a deeper depth of stimulation compared with tACS.

Furthermore, to explore the influence of TI electrical stimulation on human motor functions, Ma et al. (2021) performed TI electrical stimulation on the left primary motor cortex (M1) in healthy subjects. They designed two motor tasks, including a random reaction time task (RRTT) and a serial reaction time task (SRTT), to evaluate the motor function of humans. The envelope frequencies of TI electrical stimulation were 20 Hz (beta) and 70 Hz (high-gamma) in view of the neural oscillation related to M1. It turned out that only 70 Hz of TI electrical stimulation promoted participants’ reaction time (RT) in the RRTT experiment; meanwhile, only 20 Hz of TI electrical stimulation facilitated participants’ motor learning and increased the amplitude of motor evoked potentials in the SRTT experiment. The above findings validated the effectiveness of TI electrical stimulation on the human brain for the first time. Moreover, stimulation with different envelope frequencies demonstrated diverse effects in motor tasks, which may suggest the frequency-specific modulation of TI electrical stimulation. In addition, Zhu et al. (2022) collected the resting-state functional magnetic resonance imaging (fMRI) data of healthy subjects during TI electrical stimulation. They further verified the neuromodulation effects of TI electrical stimulation on the human motor cortex. TI electrical stimulation was found effective in boosting the functional connection strength between the primary motor cortex and the secondary motor cortex of human brains, which would increase the cortical excitability and promote the enhancement of motor function. Moreover, it was worth noting that no difference existed between TI electrical stimulation and transcranial direct-current stimulation (tDCS) in functional connectivity effects when comparing stimulation effects of both. TI electrical stimulation may have similar neuro-modulatory effects to tDCS, which is expected to become a promising intervention to improve motor learning and promote rehabilitation training in neurodegenerative disorders such as stroke and Parkinson’s disease.

Consequently, TI electrical stimulation can effectively stimulate the motor cortex and enhance motor function. In addition, given other non-invasive brain stimulation technologies are widely used to modulate cognitive function, such as perception, learning, and working memory (Miniussi et al., 2013; Yavari et al., 2018). Whether TI electrical stimulation has a similar impact on aspects of cognition is also worth exploring; this technique may become an attractive tool to study and modify cognitive processes in humans.

#### 2.3.2. Clinical applications

In addition to the regulating effects on the motor cortex in healthy subjects, TI electrical stimulation was also used to map out and stimulate pathological targets for locating epileptogenic zones and peripheral nerve stimulation (Collavini et al., 2021; Botzanowski et al., 2022).

In the surgical treatment of drug-resistant epilepsy, the physical location and number of implanted electrodes are restricted due to the complexity of brain structure (Guye et al., 2006; An et al., 2020; Frauscher, 2020). On the contrary, TI electrical stimulation could realize tunable stimulation at deep brain targets without the movement of electrodes, which might support the treatment of epilepsy. Accordingly, Missey et al. (2021) proposed a method of orientation-tunable TI electrical stimulation to identify the localization of epileptogenic zones. They utilized TI electrical stimulation with subdural electrodes to evoke seizure-like events (SLEs) in mice, and all of the mice exhibited epileptic seizure when applied TI electrical stimulation with 600 μA per electrode pair. TI electrical stimulation produced the same electrophysiological and behavioral event as implanted electrodes, demonstrating the feasibility of this minimally invasive method. In another study, Collavini et al. (2021) expected to utilize TI electrical stimulation for pre-surgical epilepsy mapping similarly. For this purpose, this study proposed to use contacts between stereo-electroencephalography (SEEG) electrodes, instead of adjacent contacts of the single electrode, to inject the stimulation current into human brains. To validate the feasibility of the method, they applied TI electrical stimulation with 10 kHz and 10.01 kHz in two different contact pairs and injected a current of 1 mA to trigger seizure in a patient. Finally, the typical spontaneous seizures were observed in an epilepsy patient, and thus TI electrical stimulation successfully stimulated the brain regions between electrodes. To sum up, TI electrical stimulation could realize focal stimulation in brain regions without more implanted electrodes, and it thus can be used for the precise location of epileptogenic focus. Significantly, this study applied TI electrical stimulation in patients’ brain for the first time, unlocking the application for probing brain function in humans.

Based on Grossman’s studies, some researchers also proposed combining TI electrical stimulation with some peripheral nerve stimulation methods to obtain better therapeutic effects. Sunshine et al. (2021) considered TI electrical stimulation as a novel modality for respiratory stimulation. The researchers firstly built rat models of drug overdose-induced respiratory depression, and then used epidural electrodes placed on the spines of rats to exert TI electrical stimulation. They found that the diaphragm muscle of the rat model contracted strongly, and the rat was able to restore breathing rapidly as the stimulation waveform of TI electrical stimulation shifted. Moreover, TI electrical stimulation was found effective in activating spinal motor neurons after spinal cord injury, which provided a new intervention for treating spinal cord injury. Here they showed the potential application of TI electrical stimulation for respiratory stimulation, further extending the clinical application of this novel approach. Botzanowski’s study tested TI electrical stimulation on the murine sciatic nerve model, and then observed obvious muscle contractions and leg movements in mice corresponding to the envelope waveforms, validating the activation of the sciatic nerve (Botzanowski et al., 2022). Additionally, TI electrical stimulation could provide more effective actuation with lower current amplitudes than normal transcutaneous electrical stimulation (TENS). Lee et al. (2021) applied TI electrical stimulation for the treatment of overactive bladder. They performed silico and *in vivo* experiments to evaluate the penetration efficiency of the proposed TI therapy. The findings showed that TI electrical stimulation could work to increase voiding volume and decrease contraction frequency of the bladder, successfully inhibiting bladder activity. Besides, a computational modeling study aimed to explore the feasibility of spatially selective retinal stimulation *via* TI-induced electric fields (Su et al., 2021). According to the modeling results, TI stimulation with appropriate electrode montages could achieve selective and focal stimulation in a specific area of retinal neurons, whereas traditional transcorneal electrical stimulation could only stimulate the peripheral area of the retina. The TI strategy effectively expanded the stimulation range, it may be a feasible strategy for spatially selective retinal stimulation. These papers display the clinical potential of TI electrical stimulation to become a promising modality for peripheral neurostimulations to some extent.

In brief, minimally invasive TI stimulation electrodes were used in most current studies, as presented in Table 1, circumventing the attenuation of stimulation waveforms due to skin, muscle, or bone. This minimally invasive interface effectively improves the stimulation intensity and focus, and it might support clinical applications of TI electrical stimulation in the future.

**TABLE 1:** The developments and applications of temporally interfering (TI) electrical stimulation.

References	Subjects	Technique	Intensity	F (kHz)	△f (Hz)	Target	Outcomes
	**Animal experiments**						
	**Animal type**	**Electrode type**	**Intensity**	**F (kHz)**	**△f (Hz)**	**Target**	**Results**
Grossman et al., 2017	Mouse	Cranial electrodes	125 μA	2	10	Hippocampus	First proposed TI electrical stimulation and successfully activated neurons in the hippocampus of mouse.
Song S. et al., 2021	Mouse	Cranial electrodes	1 mA	2	1	Superior colliculus	Successfully activated superior colliculus and caused the eye movements in mouse.
Zhang et al., 2022	Rat	Cranial electrodes	0.9 ± 0.1 mA	2	3/5/10	Primary motor cortex (M1)	Successfully activated M1 and induced related movements in rat model.
Missey et al., 2021	Mouse	Subdural electrodes	600 μA	1.2	50	CA3 of hippocampus	Used TI to evoke seizure-like events (SLEs) in mice.
Sunshine et al., 2021	Rat	Epidural electrodes	8–10 mA	5	1	Diaphragm	Used as a novel modality for respiratory stimulation after drug overdose in rat model.
Botzanowski et al., 2022	Mouse	Transcutaneous electrodes	350 μA	3	0.5–4	Sciatic nerve	Applied TI for peripheral nerve stimulation and successfully activated the motor fibers within the sciatic nerve.
Lee et al., 2021	Rat	Transcutaneous electrodes	6.4 ± 1.5 V	2	10	Bladder	Applied TI to treat the overactive bladder, demonstrating high penetration efficiency and physiological effectiveness.
	**Human trials**						
Ma et al., 2021	Healthy subjects	Transcutaneous electrodes	2 mA	2	20/70	Primary motor cortex (M1)	The first human trials, further validated the effectiveness of TI on human brain.
Collavini et al., 2021	Patient	Implanted electrodes	1 mA	10	10	Epileptogenic zones	Used TI to trigger the typical spontaneous seizures in patient.
	**Computational modeling**						
Su et al., 2021	Modeling	Extraocular electrodes	1 mA	–	–	Retina	Used TI to realize spatially selective retinal stimulation.

F, carrier frequency of TI; △f, envelop frequency.

### 2.4. Stimulation optimization

**FIGURE 2:**
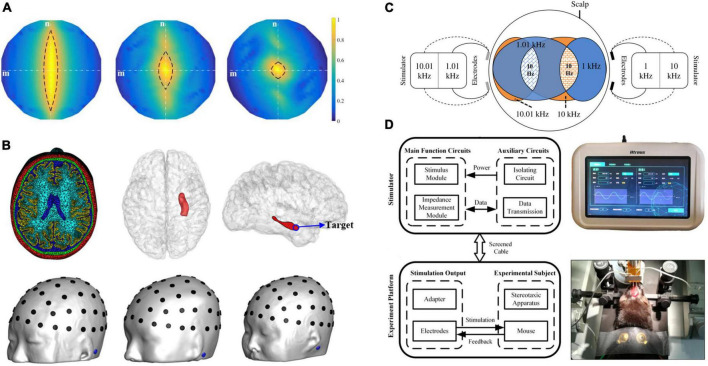
Various TI electrical stimulation optimization strategies. **(A)** Multi-electrode stimulation: the more electrode pairs employed, the more focal stimulation in the brain can be accomplished (Song X. et al., 2021). **(B)** Algorithm optimization of stimulation parameters to discover the best electrode configuration and injection current across 61 candidate electrodes on the scalp. The right hippocampal head is the stimulation target (Lee et al., 2020). **(C)** Multi-Point Temporal Interference (MTI) method: MTI used a single electrode delivering currents with different frequencies to stimulate multiple target areas in the brain simultaneously (Zhu et al., 2019). **(D)** Hardware: the stimulator could precisely position targets and measure bioimpedance in real-time during TI electrical stimulation (Wang et al., 2020).

A computational analysis demonstrated that TI electrical stimulation might not outperform other multi-electrode transcranial stimulation methods in terms of stimulation intensity (Huang and Parra, 2019). Although TI electrical stimulation has been proven effective in murine, the complexity of brain anatomical structure probably challenged the further application of TI electrical stimulation in humans (Mills et al., 2021). Therefore, efforts should be made to enhance the stimulation performance of TI electrical stimulation. Therefore, several studies have put forward various optimization strategies to maximize TI-induced electric fields in brain targets. As shown in Figure 2, this part mainly introduces the various optimization strategies from the aspects of electrode montages, optimal parameters, hardware, and other protocols (Zhu et al., 2019; Lee et al., 2020; Wang et al., 2020; Song X. et al., 2021).

#### 2.4.1. Electrode montages

To our knowledge, multi-electrode stimulation can successfully increase target intensity and focality (Dmochowski et al., 2011). Song X. et al. (2021) developed a multi-channel TI stimulation approach that employed multiple pairs of electrodes to identify smaller regions of the brain accurately. They performed computational modeling, phantom experiment and animal experiment to examine the effectiveness of the proposed strategy. The findings showed that the focality of three-channel TI and six-channel TI stimulation was improved by 54.4 and 70.2%, respectively, as compared to single-channel TI stimulation, according to computational analysis and *in vivo* studies. Furthermore, in animal experiments, the intensity of multi-channel TI stimulation was reduced by an average of 28.5%. Consequently, the multi-channel tTIS might increase stimulation focality while simultaneously reducing scalp sensation. In addition, Huang et al.’s (2020) study employed an electrode array instead of two electrode pairs to optimize modulation effects at brain targets. They used algorithms in conjunction with human head models to systematically adjust the stimulation current necessary for each electrode, resulting in improved focality. Cao and Grover (2020) proposed using a “patch-pair” made up of several electrode pairs, with each electrode pair producing electric currents with the same frequency. These patch-pairs might function as “current lenses,” improving the resolution of multi-electrode TI electrical stimulation. The use of electrode arrays was also suggested in this study; however, the same interference in different electrodes may limit further optimization.

#### 2.4.2. Optimal parameters

In the aspect of parameter optimization, some studies suggested using algorithmic optimization approaches to estimate the current intensity and electrode montage. An exhaustive algorithm was utilized in Lee et al.’s (2020) work to discover the ideal electrode configuration among 61 possible electrodes on the scalp. This algorithm identified maximum TI currents to the targeted location, which was the head of the right hippocampus. Among the three finite element head models, the current intensities at targets were less than 0.3 V/m. Despite the lack of a substantial increase in stimulation intensity, optimized TI electrical stimulation had better focality than unoptimized TI electrical stimulation or standard tACS. Furthermore, Rampersad et al. (2019) searched for the optimal four-electrode configuration among 88 electrodes by a similar exhaustive algorithm. As stimulation targets, three brain areas were chosen: the left hippocampus head, the right pallidum, and the left motor cortex. The greatest electric field intensities recorded in the three target areas were, in order, hippocampus (0.24 V/m), pallidum (0.37 V/m), and left motor cortex (0.57 V/m). This study revealed that TI electrical stimulation permitted more steerable and deeper stimulation than traditional tACS, suggesting that it might be used as an alternative or improved stimulation approach for tACS. Besides, a few studies also attempted to use more advanced algorithmic methods, such as artificial neural network (ANN) or genetic algorithm, to estimate the stimulation parameters of TI electrical stimulation, leading to more targeted stimulation on individual models (Karimi et al., 2019; Stoupis and Samaras, 2022).

In particular, because of anatomical variability in the human brain, the ideal stimulation settings for TI electrical stimulation varied between participants (Mills et al., 2021; Evans et al., 2022). The researchers examined the individual variability of the TI-induced electric fields in 25 human head models at the same stimulation level in a recent study (von Conta et al., 2021). The electric field distribution may be affected by anatomical differences between human participants. Individual participants’ TI-induced electric fields in the three target areas (left hippocampus, left motor cortex, and thalamus) were shown to be varied. The findings indicated that tailored parameter adjustments might increase the efficacy of TI electrical stimulation. Given inter-individual variability in human brains, a precise and individual optimization strategy benefits achieving more concentrated and effective stimulation. Therefore, individual stimulation strategies can be used to improve the stimulation performance of TI electrical stimulation.

#### 2.4.3. Hardware

To get a high-precision output of TI electrical stimulation, Wang et al. (2020) created a TI stimulator that can accurately position targets and detect bioimpedance online. In this study, they not only employed an analog phase accumulator to improve the precision of stimulation waveforms but also exploited anti-phase current drive technology to reduce crosstalk across channels and then keep the independence of the channel outputs. Afterward, a mouse experiment was conducted to test the output performance of this stimulator. The experiments proved the TI stimulator’s dependability and position precision, which could successfully activate neurons under the motor cortex. Zhang et al. (2022) further developed an integrated TI device for animal brain stimulation. This device differently used a direct digital synthesizer (DDS) to output stimulation waveforms and a current transformer to eliminate the current crosstalk between channels. And the bioimpedance could be also measured by the stimulation potential detector. This TI device was tested in live rats and successfully stimulated the rat’s motor cortex. During stimulation, the device can precisely produce sinusoidal currents and monitor bioimpedance, confirming its feasibility and safety. Both researches aimed to assist users in achieving safe and precise TI electrical stimulation without the need for further modeling and simulation. Furthermore, Ahsan et al. (2022) constructively developed a minimally invasive TI electrical stimulation system, which used gigahertz (GHz) electromagnetic waves delivered by endocranial antenna arrays for deep brain stimulation. The computer modeling results revealed that utilizing two endocranially implanted arrays of size 4.2 cm 4.7 cm each, an intensity of 12 V/m with a focality of 3.6 cm at a deep brain target could be achieved. This approach significantly improved the spatial resolution of TI electrical stimulation and is expected to be used to stimulate deep brain targets, although it may require additional experimental validation for further applicability.

#### 2.4.4. Novel protocols

Here are some more optimized TI electrical stimulation strategies. Zhu et al. (2019) pioneered the concept of Multi-Point Temporal Interference (MTI), which can simultaneously stimulate multiple target areas in the brain. Unlike multi-electrode stimulation approaches, MTI used a single electrode to provide varying frequencies of current to generate multiple stimulation sites, and it could activate several nodes in the brain network at the same time. The researchers demonstrated that MTI provided controlled and independent stimulation using a tissue phantom and a human head model. In another work, researchers used phase modulation to convert the sinusoidal envelope waveform into a pulse-like waveform to accurately regulate the stimulation time of TI electrical stimulation (Terasawa et al., 2022). This method generated more accurate and steerable TI envelope waveforms than standard TI electrical stimulation, boosting the usability of this technology.

## 3. Future directions

**FIGURE 3:**
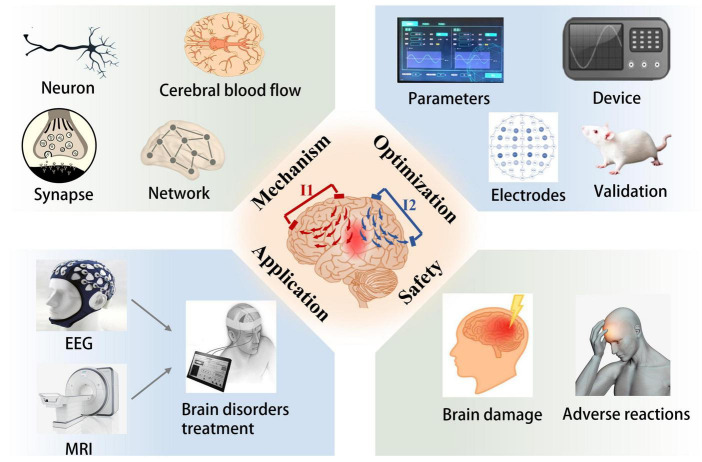
Potential future directions for TI electrical stimulation, with four factors to consider: mechanism, optimization, application, and safety. The subgraph introduces the implicated or potential study direction of TI electrical stimulation.

The non-invasive brain stimulation techniques have emerged as clinically available options for the diagnosis and treatment of brain disorders for decades (Hoy and Fitzgerald, 2010; Boes et al., 2018; Walther and Baeken, 2021). As a novel non-invasive brain stimulation modality, TI electrical stimulation holds the advantages of increased spatial specificity and depth selectivity in comparison to common non-invasive brain stimulation techniques (Rampersad et al., 2019). It is expected to use for the treatment of neurological or psychiatric disorders in the future by targeting the pathological circuit of brain tissue (Grossman et al., 2018). However, most current studies about TI electrical stimulation are limited to computational modeling and numerical simulation, and a few results of these studies have only been verified in rodents (Grossman et al., 2017). Researches on TI electrical stimulation are still in the early stages which need further exploration (Bouthour et al., 2017; Dmochowski and Bikson, 2017). As displayed in Figure 3, more studies are required to explore the mechanisms, optimal stimulation protocols, as well as therapeutic applications. For example, using neuron models and animal experiments to understand the basic neurophysiological mechanisms; employing multi-electrodes method and optimization algorithms to enhance stimulation efficiency. The next will introduce the potential future directions of TI electrical stimulation.

### 3.1. Mechanism

According to previous studies, TI electrical stimulation may provide subthreshold neuromodulation rather than direct recruitment at lower field strengths, which seems similar to tACS (Rampersad et al., 2019; Esmaeilpour et al., 2021; Howell and McIntyre, 2021). As far as we know, both tACS and TI electrical stimulation employ low-frequency alternating current to regulate the brain targets. In tACS, the currents applied to brain tissue can cause the polarization of neuronal cell membranes and in turn, alter the neural excitability of brains (Liu et al., 2018). Previous studies have found the neurophysiological effects of tACS include online effects and offline effects (Yavari et al., 2018). Online effects refer to the changes in excitability of neurons that occur acutely during stimulation (Elyamany et al., 2021). For instance, the nerve entrainment of tACS can induce endogenous neural oscillations in the brain (Ahn et al., 2019). On the contrary, offline effects refer to the alterations in neural plasticity, including glial cells, the immune system, cerebral blood flow, neural networks, and so on, triggering long-term stimulation effects outlast the stimulation (Bachinger et al., 2017; Cirillo et al., 2017). In specific, von Conta et al. (2022) initially investigated neural entrainment of TI electrical stimulation to alpha oscillations. Although the results were not ideal, there was no significant difference between TI electrical stimulation and control stimulation; subsequent studies could consider the neural oscillations at different frequencies and further perfect the experimental methods. All in all, future studies should focus on whether TI electrical stimulation have similar effects to tACS or other non-invasive neuromodulation technologies, which may involve acute changes of neural activity during stimulation as well as long-lasting alterations of synaptic plasticity (Yavari et al., 2018).

### 3.2. Optimization

Accurate and effective stimulation in brain targets is essential for the application of brain stimulation techniques (Guler et al., 2016). Several studies have investigated the electrical field distribution of TI electrical stimulation in human brains. However, most of the studies used human head models for optimization analysis while no corresponding *in vivo* experiments for validation. And the findings indicated that the stimulation intensity of TI electrical stimulation was not significantly higher than that of TES (Huang and Parra, 2019; Rampersad et al., 2019). The low electric field strength was considered a limiting factor for TI electrical stimulation. To overcome these barriers, more advanced optimization strategies can be used to maximize the electrical fields at brain targets while minimizing superficial stimulation and undesired effects (Huang and Parra, 2019). For instance, multi-electrode stimulation can enhance the electric field intensity and focality at targets. Optimization algorithms help customize individual stimulation protocols by determining optimal electrode placement and injected currents (Lee et al., 2020; Song X. et al., 2021). Additionally, as a valuable research means, animal models can be applied in subsequent studies to evaluate the related optimized strategies as well. At last, some brain disorders may require continuous stimulation to achieve therapeutic benefits. Hence the development of a specialized TI-delivering device is necessary in the future (Lozano, 2017).

### 3.3. Application

Multiple anatomical and experimental studies have evidenced that the significant differences in the brain anatomical structures between experimental animals and humans, the size of human brain is approximately 1,400 times larger than the mouse brain. For this reason, human brains may need a larger current intensity to achieve similar stimulation effect comparable to that of experimental animals, which limits the potential applications of non-invasive brain stimulation technologies to some degree (Lozano, 2017). At present, TI electrical stimulation has only successfully stimulated the hippocampus of mice with minimally invasive electrodes (Grossman et al., 2017), it is not clear whether TI electrical stimulation can be extended to human brains. In order to further verify the utility of TI electrical stimulation, electroencephalography (EEG), functional magnetic resonance imaging (fMRI), and other technologies can be used to explore the brain activity in humans during stimulation. Moreover, some experts pointed out that stroke, obsessive-compulsive disorder, epilepsy, depression or spinal cord injury may be attractive initial indications for TI electrical stimulation (Grossman et al., 2018). It is because that the pathological targets of these disorders are relatively deep whereas common non-invasive brain stimulation techniques are not easy to reach (Bouthour et al., 2017). There will be a promising issue to investigate the application of TI electrical stimulation in disease models.

### 3.4. Safety

The assessment of safety and tolerability will be an essential issue if developing TI electrical stimulation into a new therapeutic modality. The safety of TI electrical stimulation generally includes two aspects, that is, adverse reactions of the subjects as well as possible damage to deep brain structures and neurons (Grossman et al., 2017). A study evaluated subjects’ adverse reactions after applying TI electrical stimulation of 2 mA, and found that among 100 subjects, only 4 subjects behaved adverse reactions like fatigue and dizziness (Ma et al., 2021). In particular, McCreery’s study demonstrated that electrical stimulation at current densities less than 25 mA/cm^2^ would not damage brain tissue (McCreery et al., 1990). The electric current intensity applied in TI electrical stimulation is approximately 1–2 mA, and the electric field generated in the brain is less than 1 V/m, which is within the safe limits.

Grossman et al. (2017) examined the changes of different molecular mediators, such as neurons, glial cells, and synaptic molecules, to evaluate possible brain injury during TI electrical stimulation. The results showed that the quantity and morphology of neurons or synapses do not alter after stimulation, which verified the safety of TI electrical stimulation in brain tissue. Unfortunately, recent studies found the phenomenon of high-frequency conduction block with TI electrical stimulation, which may affect off-target neurons and cause undesired side effects and restrict (Mirzakhalili et al., 2020). Overall, future studies should consider the safety limits of TI electrical stimulation, and computational models and animal experiments could be used to define safety standards.

## 4. Conclusion

With the development of non-invasively activating neurons at deep sites, TI electrical stimulation has attracted growing attention. The ultimate purpose of TI electrical stimulation is to modulate neuronal activity in deep brain regions, hence assisting with the therapy of brain disorders. The preliminary studies discovered that TI electrical stimulation could not match the efficacy of DBS in terms of stimulation intensity and focality (Grossman et al., 2018). However, because of the comparable electric field intensity and better focality, it may be used as an alternative or improved stimulation approach for traditional TES. Furthermore, considering the considerable anatomical differences between animal models and humans, whether the findings in mice could be translated to human beings requires further evaluation.

## Author contributions

All authors contributed to the manuscript revision, read, and approved the submitted version.
